# Reduced expression of the presynaptic co-chaperone cysteine string protein alpha (CSPα) does not exacerbate experimentally-induced ME7 prion disease

**DOI:** 10.1016/j.neulet.2015.01.053

**Published:** 2015-03-04

**Authors:** Matthew J. Davies, Matthew Cooper, V. Hugh Perry, Vincent O’Connor

**Affiliations:** Centre for Biological Sciences (CfBS), University of Southampton, Southampton SO17 1BJ, United Kingdom

**Keywords:** CSPα, Gliosis, ME7, PrP^Sc^, Synaptic loss

## Abstract

Infection of mice with the ME7 prion agent results in well-characterised neuropathological changes, which includes vacuolation, neurodegeneration and synaptic degeneration. Presynaptic dysfunction and degeneration is apparent through the progressive reduction in synaptic vesicle proteins and eventual loss of synapses. Cysteine string protein alpha (CSPα), which regulates refolding pathways at the synapse, exhibits an early decline during chronic neurodegeneration implicating it as a mediator of disease mechanisms. CSPα null mice develop a progressive neuronal dysfunction through disruption of the integrity of presynaptic function. In this study, we investigated whether reduced expression of CSPα would exacerbate ME7 prion disease. Wild type (+/+) and heterozygous (+/−) mice, which express about a ∼50% reduction in CSPα, were used as a distinct genetic background on which to impose prion disease. +/+ and +/ − mice were inoculated with brain homogenate from either a normal mouse brain (NBH) or from the brain of a mouse which displayed clinical signs of prion disease (ME7). Behavioural tests, western blotting and immunohistochemistry, which resolve key elements of synaptic dysfunction, were used to assess the effect of reduced CSPα on disease. Behavioural tests revealed no change in the progression of disease in ME7–CSPα +/− animals compared to ME7–CSPα +/+ animals. In addition, the accumulation of misfolded PrP^Sc^, the diseased associated gliosis or synaptic loss were not different. Thus, the misfolding events that generate synaptic dysfunction and lead to synaptic loss are unlikely to be mediated by a disease associated decrease in the refolding pathways associated with CSPα.

## Highlights

• CSPα is reduced in ME7-animals during disease progression.
• CSPα heterozygosity does not accelerate behavioural changes in ME7-animals.
• Prion disease pathology is not altered by reduced CSPα expression.

## 1 Introduction

Prion diseases, are a group of rare and fatal neurodegenerative diseases of human and animals [1] involving the conversion of the cellular prion protein (PrP^c^) into a misfolded form (PrP^Sc^), which accumulates and deposits as amyloid plaques [2]. Characteristics of these diseases include gliosis, spongiform changes, and synaptic loss which proceeds neuronal death [3–6]. Prion diseases can occur sporadically, be genetically inherited or transmitted infectiously [7]. This infectious capacity, unique amongst neurodegenerative diseases, was successfully exploited in the development of models of chronic neurodegeneration [8,9]. Pathology in prion-infected mice develops in a well-defined and predictable manner, over a time course dependent on the prion strain used. This well-defined temporal progression renders prion-based models ideal for investigating significant disease events and underlying mechanisms of pathology [5,6,10,11].

One murine model, utilising the ME7 prion agent, involves bilateral injection of ME7-infected brain homogenate into the dorsal hippocampus of C57BL/6J mice [5] and this paradigm leads to hippocampal pathology shared by several strains [12,13]. This sequence of progression includes PrP^Sc^ deposits, hypertrophied astrocytes and activated microglia, followed shortly after by synaptic loss in the stratum radiatum of the hippocampus [5,6]. However, neuronal loss is not seen until late-stage disease [5]. The early synaptic loss appears to selectively involve the presynaptic compartment, with reduced expression of a number of presynaptic proteins [6]. One such synaptic protein which shows an early and progressive reduction in the hippocampus of ME7-animals is CSPα [6] and in view of its role in synaptic re-folding suggests potential for a direct role in disease progression.

CSPα is a synaptic vesicle protein and functions as a molecular chaperone in conjunction with Hsc70 and a small glutamine-rich tetratricopeptide repeat (TRP)-containing protein (SGT) [14] which controls the conformational folding of the SNARE protein, SNAP-25 [15]. CSPα null mice are normal at birth but develop a progressive muscle weakness and sensorimotor deficit between 2 and 4 weeks of age [16]. At about ∼P15 these mice stop gaining weight, become lethargic and begin to die in the second postnatal month [16]. There is however, no obvious difference between wildtype (+/+) and heterozygous (+/−) CSPα mice, which suggests that reduced levels (∼50%) of CSPα is not sufficient to cause a neurodegenerative phenotype. In contrast, mutations that reduce the human CSPα gene DNAJC5’s function cause ceroid-lipofuscinosis that coincides with accelerated age-dependent neurodegeneration [17]. Finally, CSPα and associated chaperone activities are also more widely implicated in proteostasis [18]. This led us to reason that the ME7 prion disease pathology would, via direct synaptic dysfunction or deficient proteostasis, be exacerbated in CSPα +/− mice. To test this we used behavioural assays and molecular changes that act as sensitive measures of disease evolution in cohorts of CSPα +/+ and CSPα +/− animals infected with ME7.

## 2 Materials and methods

### 2.1 Animal husbandry

CSPα +/+ and +/− mice were generated as described [16,19,20] and crossed and maintained on a C57BL/6J Charles River background [21]. The cohort of 26 animals (13 CSPα +/+ and 13 CSPα +/−) used in this study were generated from a common set of littermates. All animals were housed according to UK Home office regulations, on a standard 12 h: 12 h light–dark cycle at an ambient room temperature of 21 ± 2 °C, with food and water provided *ad libitum*.

### 2.2 ME7 prion disease

All procedures were carried out under a UK Home Office licence and in accordance with the United Kingdom Animals (Scientific Procedures) Act, 1986. Surgical procedures were carried out as previously described [5]. CSPα +/+ and +/− female animals 8–13 weeks of age were anaesthetized and bilaterally injected into the dorsal hippocampus with either 1 μl of NBH or ME7 homogenate, using the stereotaxic co-ordinates anteroposterior +2.0 mm, lateral ±1.7 mm and depth −1.6 mm measured at Bregma. Eleven weeks post-inoculation (w.p.i.) NBH- and ME7-animals were subjected to behavioural tests, widely used to define preclinical and clinical disease. These tests included burrowing, glucose consumption and open field as measure of affective behaviour as previously described [5,10,21]. In addition, muscle strength and co-ordination were measured using an inverted screen as described previously [10,21]. The experiments followed a schedule of inverted screen tests preceding early afternoon open field tests, followed by late afternoon two hour burrowing tests. 24 h burrowing was then tested overnight in conjunction with glucose consumption. All animals used in the study were killed at a humane endpoint at 21 w.p.i. regardless of treatment or genetic background. At this point all animals were terminally anaesthetized with sodium pentobarbital and perfused transcardially with heparinised saline.

### 2.3 General tissue processing

For western blotting hippocampal tissue was micro-dissected on dry ice as described [21]. Brain tissue for immunohistochemistry was perfused and post-fixed with 10% neutral buffered formalin and subsequently paraffin-embedded as detailed elsewhere [6].

### 2.4 Western blotting

The dissected hippocampi from animals sacrificed at 21 w.p.i. were homogenised in 5 volumes (w/v) of RNase-free 1 × PBS supplemented with a protease and phosphatase inhibitor cocktail (Thermo Scientific). Each hippocampal homogenate was combined with an equal volume of lysis buffer (40 mM HEPES pH 7.4, 250 mM NaCl, 4% v/v SDS supplemented with a protease and phosphatase inhibitor cocktail (Thermo Scientific)). Samples were heated at 95 °C and subsequently centrifuged. The supernatant was collected and the protein concentration determined using the Bio-Rad Dc protein assay (Bio-Rad). Hippocampal homogenates were then diluted equivalently. Equal amounts of protein were resolved by SDS-PAGE and subjected to fluorescent-based western blotting or stained with colloidal Coomassie Blue [6]. Following blocking in 5% w/v non-fat milk, nitrocellulose membranes were incubated in 5% w/v bovine serum albumin (BSA) containing 0.1% v/v Tween-20 and one of the following primary antibodies: anti-CSPα (1:1,000; Abcam); anti-GFAP (1:5,000; Dako); anti-PrP (1:5,000; 6H4 Prionics); anti-Synapsin (1:1,000; Chemicon); anti-Synaptophsyin (1:1,000; Abcam) and anti-VAMP-2 (1:1,000; Synaptic Systems). Membranes were then probed with the appropriate fluorescent-coupled goat anti-mouse or anti-rabbit secondary antibody (Licor). Immunoreactivity of protein bands was determined using a Licor Odyssey infrared detection system (Licor). The signal obtained for each antigen was normalised to total protein, as measured by the signal obtained from scanning individual lanes of colloidal Coomassie stained gels.

### 2.5 Immunohistochemistry

10 μm paraffin-embedded coronal hippocampal sections were cut on a microtome, and subsequently dewaxed in xylene and rehydrated through a decreasing series of ethanol concentrations. Non-specific endogenous peroxidase activity was eliminated by incubation with 1% H_2_O_2_ and antigen retrieval was performed using citrate buffer (pH 6) and microwaving, or autoclaving-formic acid treatment for PrP^Sc^ [5,6]. Non-specific antibody binding was blocked by incubation with the appropriate serum. Subsequently, sections were incubated in a humid chamber with one of the following primary antibodies: anti-GFAP (1:1000; Dako), anti-IBA1 (1:500; Abcam); anti-PrP (1:4000; 6H4 Prionics) and anti-Synaptophysin (1:100; Abcam). Specific binding was detected using a biotinylated secondary antibody (Vector Laboratories), followed by incubation in ABC (Vector Laboratories) and visualisation using DAB. Nuclei were counterstained with Harris hematoxylin.

### 2.6 Statistical analysis

For behavioural tests, repeated measures two-way ANOVA was used with Bonferroni post-analysis. Unpaired *t*-test was used for biochemical data. The statistical analysis was performed using Graph Pad Prism (version 6, Graph Pad Software Inc.). Quantification values were expressed as the mean ± standard error of the mean (S.E.M.), with a *p* value of ≤0.05 considered as statistically significant. Behavioural tests, *n* = 4 (NBH) and *n* = 8 (ME7); western blotting, *n* = 3 (NBH) and *n* = 4 (ME7) and immunohistochemistry, *n* = 2 (NBH and ME7).

## 3 Results

### 3.1 Reduced expression of CSPα does not exacerbate behavioural changes in ME7-animals

**Fig. 1:**
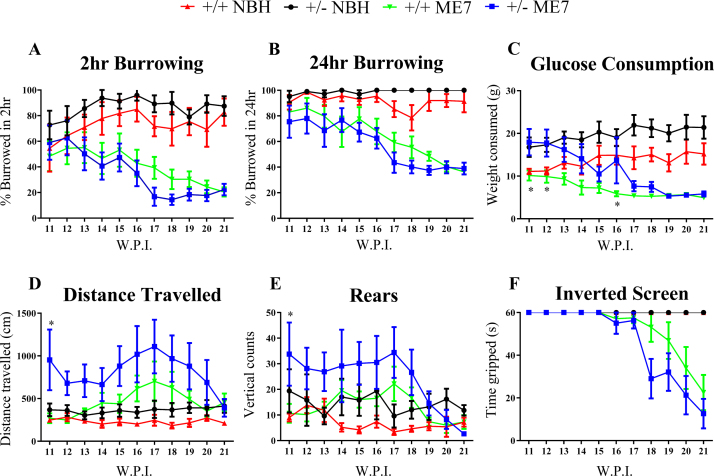
Behavioural changes in CSPα +/+ and +/− NBH- and ME7-animals. Burrowing behaviour (A and B), glucose consumption (C), distance travelled (D), rears (E) and inverted screen strength (F) were tested. There were no significant differences in the behaviours between CSPα +/+ or +/− animals infected with ME7. The baseline levels for CSPα +/− animals are higher for burrowing, glucose consumption, distance travelled and rears compared to CSPα +/+ animals. Data in graphs represents mean ± /SEM from *n* = 4 animals (NBH) and *n* = 8 animals (ME7). *P ≤ 0.05, repeated measures two-way ANOVA with Bonferonni post-analysis. +/+, wildtype; +/−, heterozygous.

Previous behavioural studies in ME7-animals show a progressive decrease from 12 w.p.i. onwards in the number of pellets burrowed compared to NBH-animals, concurrent with a decrease in glucose consumption and an increase in distance travelled and rears [5,10,21]. Additionally, at 18 w.p.i., motor deficits become apparent, as evidenced by declining performance in the inverted screen test [10,21]. This decline in behavioural performance as a consequence of prion disease is apparent in the behavioural tests performed as part of this study, with both ME7–CSPα +/+ and +/− animals showing progressively decreasing burrowing behaviour (Fig. 1A and B) and glucose consumption (Fig. 1C), increased distance travelled (Fig. 1D) and rears (Fig. 1E) and reduced strength (Fig. 1F) compared to NBH-animals. Although CSPα +/− animals have a higher baseline level in the number of pellets burrowed in 2 h (Fig. 1A) and overnight (Fig. 1B), the amount of glucose consumed (Fig. 1C), distance travelled (Fig. 1D) and rears (Fig. 1E), there was no difference in the progression of the behavioural decline in ME7-animals between CSPα genotypes (Fig. 1A–F).

Protein expression of markers of prion pathology reveals no difference between CSPα +/+ and +/− animals infected with ME7.

**Fig. 2:**
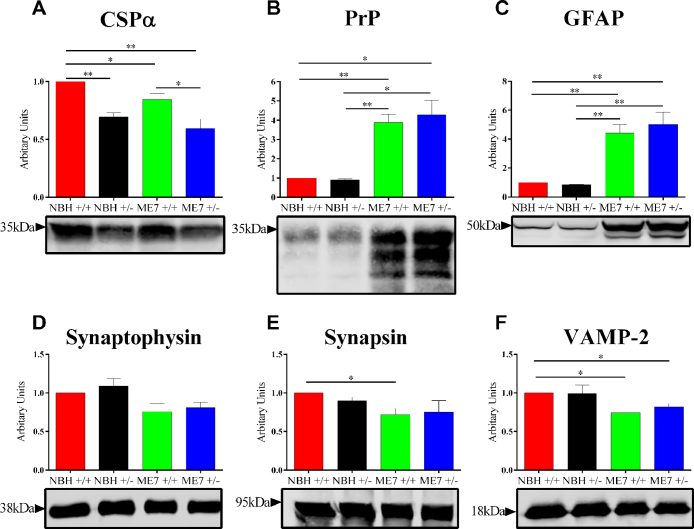
Analysis of prion pathology in ME7–CSPα +/+ and +/− animals. Quantitative western blotting of CSPα (A), total PrP (B), GFAP (C), Synaptophysin (D), Synapsin (E) and VAMP-2 (F) in hippocampal homogenates from CSPα +/+ and +/− mice inoculated with either NBH or ME7. Representative western blots are shown. (A) A decrease in CSPα expression is seen in +/− animals compared to +/+. CSPα expression is further reduced in ME7-animals compared to NBH-animals. (B) Significant differences in total PrP immunoreactivity were seen between NBH- and ME7-animals, but no difference was seen between CSPα +/+ and +/− animals injected with ME7. (C) ME7 infection causes increased expression of the astrocyte marker GFAP. However, there is no difference in expression between ME7–CSPα +/+ and +/− animals. (D–F) The levels of the three presynaptic proteins Synaptophysin (D), Synapsin (E) and VAMP-2 (F) are reduced in ME7-animals compared to NBH-animals. There is no change in the expression of these three proteins between ME7–CSPα +/+ and +/− animals. Data in bar charts represents mean ± SEM from *n* = 3 animals (NBH) and *n* = 4 animals (ME7). *P < 0.05 and **P <0.01, unpaired *t*-test. +/+, wildtype; +/−, heterozygous.

Hippocampi taken from brains extracted at 21 w.p.i. were homogenised and used for western blotting to study expression levels of CSPα (Fig. 2A), total PrP (Fig. 2B), the astrocyte marker GFAP (Fig. 2C) and the presynaptic proteins Synaptophysin (Fig. 2D), Synapsin (Fig. 2E) and VAMP-2 (Fig. 2F). Western blots for CSPα showed that CSPα +/− animals (Fig. 2A) displayed a ∼50% reduction in protein as a consequence of their heterozygous genetic background. In contrast, there are no differences in the expression of any of the other three presynaptic proteins (Fig. 2D–F) between CSPα +/+ and +/− NBH animals. This indicates that the reduced level of CSPα is not due to a decrease in the number of synaptic vesicles but rather a fall in the complement of CSPα molecules per vesicle.

As shown in previous work there was a decrease in CSPα levels during disease [6]. This is seen when comparing relative levels of the CSPα in ME7-animals compared to CSPα +/+ and CSPα +/− NBH-animals. In the latter case a decrease from an already reduced level of CSPα. Similar measurements of the presynaptic proteins Synaptophysin (Fig. 2D), Synapsin (Fig. 2E) and VAMP-2 (Fig. 2F) showed the reduced levels in ME7-animals compared to NBH-animals in both CSPα genotypes. Consistent with previous observations, the robustness of the presynaptic protein reduction due to ME7 was more marked for Synapsin and VAMP-2 [6,22].

Total PrP immunoreactivity (Fig. 2B) acts to indicate ME7 infection and prion disease development. There was a significant increase in PrP expression of un-, mono- and diglycosylated forms in both CSPα +/+ and +/− animals infected with ME7- compared to NBH-animals (Fig. 2B). However, there is no significant difference seen in its expression between ME7- and CSPα +/+ and +/− animals (Fig. 2B). Our previous data indicates that ME7 related increase in total prion immunoreactivity is a good correlate of misfolded protein [6]. Western blotting of GFAP showed an increase in its levels in ME7-animals compared to NBH-animals (Fig. 2C). However, like PrP, there was no significant difference in levels of its expression levels between ME7–CSPα +/+ and +/− animals (Fig. 2C).

**Fig. 3:**
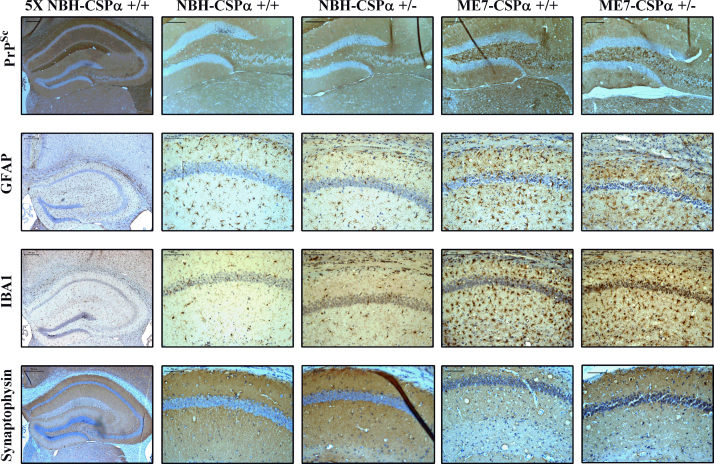
Immunostaining of hippocampal coronal sections from NBH- and ME7-injected CSPα +/+ and +/− animals. ME7 animals show PrP^Sc^ deposition in the hilus of the dentate gyrus extending to the CA3 region, increased number and size of both astrocytes and microglia and loss of synapses in the stratum radiatum of the hippocampus compared to NBH-animals. However, there are no visible differences in any of these pathologies between ME7–CSPα +/− animals compared to ME7–CSPα +/+. *n* = 2 animals per genotype and condition. Scale bars, 100 μm except for Synaptophysin images where scale bars are 200 μm and 5× images where scale bars are 300 μm. +/+, wildtype; +/−, heterozygous; CA3, Cornu Ammonis region 3.

We then performed immunohistochemistry to determine if there were any discernible changes in protein expression of some of these markers in different regions of the hippocampus. Coronal sections containing the hippocampus were taken from NBH- and ME7-animals at 21 w.p.i. The sections were immunostained for PrP^Sc^, GFAP, the microglia marker IBA1 and Synaptophysin (Fig. 3). Whilst there is no PrP^Sc^ deposition in the hippocampus of NBH-animals, we observe the appearance of these formic acid resistant deposits of PrP^Sc^, in both ME7–CSPα +/+ and +/− animals (Fig. 3). However, there is no visible difference in the number or pattern of deposition of PrP^Sc^ between CSPα +/+ and +/− animals. In ME7 hippocampi, GFAP+ astrocytes are generally larger in number, with more developed processes than in NBH-animals and often show infiltration of the neuronal layers (Fig. 3). Similarly, we observe a large increase in the number of visible microglia in ME7-animals compared to NBH-animals (Fig. 3). In addition, like astrocytes we also note the increased infiltration of microglia into the neuronal layers of the hippocampus in ME7-animals (Fig. 3). Despite this there is no clear difference in the number or appearance of astrocytes or microglia between ME7–CSPα +/+ and +/− animals. Previous studies, staining for the synaptic protein Synaptophysin revealed disorganized and a relative reduced intensity of staining in the stratum radiatum of the hippocampus as ME7 pathology progresses [5,6]. In keeping with these findings, our work revealed reduced and disorganised Synaptophysin staining in the stratum radiatum in ME7-infected brains 21 w.p.i. (Fig. 3). However, once again, there was no clear difference between ME7 and CSPα +/− animals compared to ME7–CSPα +/+ animals (Fig. 3).

## 4 Discussion

Experimentally induced ME7 prion disease presents a predictable and well-defined neuropathology enabling the correlation of cellular and molecular findings with important pathological events [5]. For example, the loss of synapses in the stratum radiatum of the hippocampus coincides with the onset of subtle behavioural changes in animals injected with ME7. In this study, we have used an established battery of behavioural tests [5,10,21] that resolve underlying pathological mechanisms at the level of the whole organism. In particular, we have investigated if the behavioural decline that marks synaptic dysfunction and mid-stage disease (burrowing, glucose consumption and open field) or late stage disease (inverted screen) differs in genetic backgrounds with different levels of CSPα.

As synaptic degeneration may be a potentially reversible event in neurodegenerative diseases, significant research has gone into discovering molecular pathways related to synaptic pathology. We have previously reported the decreased expression of a number of presynaptic proteins in ME7-animals including the synaptic chaperone CSPα [6]. CSPα knockout mice undergo premature death, however, animals with only a ∼50% reduction in CSPα levels appear comparatively normal compared to +/+ animals [16]. This indicates that a ∼50% reduction in CSPα is sufficient to largely preserve synaptic integrity. However, it is unclear at what point reduced CSPα expression becomes pathological as the complete knock out of the gene is post-embryonic lethal [16]. The focus of our study was to determine whether a genetic background of low CSPα expression would exacerbate experimentally-induced ME7 prion disease. To achieve this, we injected CSPα +/+ and +/− mice with either NBH or ME7-infected brain homogenate, and evaluated the genetic impact upon prion disease progression via behavioural tests and protein expression using western blotting and immunohistochemistry.

Given the role of CSPα in preserving synaptic function, it was hypothesized that the reduced levels of CSPα in CSPα +/− mice may increase synaptic susceptibility to degeneration and in doing so amplify the behavioural changes associated with disease. There are reports of small changes in measures of spontaneous locomotion associated with the reduction in CSPα expression in the heterozygous mice, however, this is not due to any synaptic loss [16]. This may underlie the shifted baseline behaviour we noted that was particularly clear in the glucose consumption test. However, the clear observation is that the decreased expression of CSPα did not have a significant effect in the behaviours tested between ME7 and CSPα +/+ and +/− animals (Fig. 1). These results indicate that reduced CSPα levels in +/− animals are not sufficient to accelerate disease progression.

Western blots revealed reduced levels of CSPα in CSPα +/− animals compared to +/+, with a further reduction in ME7-animals (Fig. 2A). There are estimated to be around ∼2 copies of CSPα per synaptic vesicle [23]. This would suggest that in the CSPα +/− animals where there is a ∼50% reduction of CSPα, there would only be around ∼1 copy of CSPα per synaptic vesicle, as there is no evidence for a reduced synaptic vesicle number in the CSPα +/− mice [16]. The further reduction of CSPα seen in ME7–CSPα +/− animals is likely to be due to the synaptic loss which occurs in ME7 [5,6,24]. Overt loss of synapses would reduce the content of synaptic vesicle proteins as we see in the current study. However, the previously reported differential loss of presynaptic proteins and the accumulating dysmorphic nature of synaptic vesicle profiles identified in disease could imply routes to reduced synaptic vesicle content prior to a more overt synaptic loss.

Although ME7-animals had high levels of total PrP (Fig. 2B) and deposits of PrP^Sc^ (Fig. 3) there were no significant differences in the levels of PrP or the number or distribution of PrP^Sc^ deposits between ME7 and CSPα +/+ and ME7 and CSPα +/− animals. In addition, there was no difference between the expression of GFAP (Fig. 2C), the number of astrocytes and microglia and their appearance (Fig. 3) between ME7 and CSPα +/+ and ME7–CSPα +/− animals. Despite this the most likely detrimental effect of reduced CSPα levels in ME7-animals is reduced synaptic number, given the protein’s role in chaperoning SNAP-25 and promoting vesicle exocytosis [25]. The levels of the presynaptic proteins Synaptophysin (Fig. 2D), Synapsin (Fig. 2 E) and VAMP-2 (Fig. 2F) were reduced in ME7-animals compared to NBH-animals, however, there was no difference in their expression between ME7–CSPα +/+ and +/− animals. Additionally, staining for Synaptophysin (Fig. 3) failed to reveal a difference between ME7-animals of both CSPα genotypes. This indicates that whatever mechanisms are contributing to synaptic loss seen in ME7-animals, reduction in CSPα levels is not a critical dose limiting step. Therefore, whilst a complete absence of the protein is detrimental to synaptic health, a compound reduction resulting from a heterozygous genetic background is insufficient to exacerbate ME7 prion disease.

One explanation for the apparent lack of effect of CSPα reduction on ME7 prion disease progression may be the neuronal type undergoing synaptic degeneration. In CSPα null mice, the synapses most strongly affected are those with high activity, necessitating superior SNARE function [16,26,27]. Previous prion studies proposed that highly active GABAergic synapses may undergo degeneration, as there is evidence for severe, selective GABAergic cell loss in human and experimental Creutzfeldt–Jakob disease [28,29]. However, studies in ME7 prion disease revealed no significant loss of parvalbumin (PV)-positive GABAergic inhibitory neurons in the hippocampus of ME7-animals [30]. In ME7 prion disease synaptic loss in the hippocampus occurs along the Schaffer Collateral axons of CA3 pyramidal neurons, which have lower activity and hence demand for proper SNARE chaperoning. As such, these synapses may be less susceptible to low CSPα levels than others.

## 5 Conclusion

Protein expression studies and behavioural assays of disease progression both failed to provide evidence for an effect of a CSPα-deficient genetic background on the protein misfolding or subsequent progression of prion pathology resulting from ME7 infection. This is despite the previously reported detrimental neurological consequences of CSPα absence and reports of reduced CSPα expression in ME7-infected animals. These results suggest that reducing CSPα expression to about ∼50% is not sufficient to enhance synaptic loss and prion disease pathology.

## Author contributions

M.J.D. and M.C. did most of the experimental work. V.H.P. and V.O.C. directed and supervised the project. All authors contributed to the writing of the paper.
